# The Chemokine-Based Peptide, CXCL9(74-103), Inhibits Angiogenesis by Blocking Heparan Sulfate Proteoglycan-Mediated Signaling of Multiple Endothelial Growth Factors

**DOI:** 10.3390/cancers13205090

**Published:** 2021-10-12

**Authors:** Alexandra De Zutter, Helena Crijns, Nele Berghmans, Melissa García-Caballero, Lotte Vanbrabant, Noëmie Pörtner, Vincent Vanheule, Paulien Verscheure, Mohammad Mairaj Siddiquei, Ahmed M. Abu El-Asrar, Peter Carmeliet, Pieter Van Wielendaele, Ingrid De Meester, Jo Van Damme, Paul Proost, Sofie Struyf

**Affiliations:** 1 Laboratory of Molecular Immunology, Department of Microbiology, Immunology and Transplantation, Rega Institute for Medical Research, KU Leuven, 3000 Leuven, Belgium; alexandra.dezutter@kuleuven.be (A.D.Z.); helena.crijns@kuleuven.be (H.C.); nele.berghmans@kuleuven.be (N.B.); lotte.vanbrabant@kuleuven.be (L.V.); noemie.portner@kuleuven.be (N.P.); vincent.vanheule@gmail.com (V.V.); paulien.verscheure@kuleuven.be (P.V.); jo.vandamme@kuleuven.be (J.V.D.); paul.proost@kuleuven.be (P.P.); 2 Laboratory of Angiogenesis and Vascular Metabolism, Center for Cancer Biology (CCB), VIB, Department of Oncology, Leuven Cancer Institute, KU Leuven, 3000 Leuven, Belgium; melissa@uma.es (M.G.-C.); peter.carmeliet@kuleuven.be (P.C.); 3 Department of Ophthalmology, College of Medicine, King Saud University, P.O. Box 245, Riyadh 11411, Saudi Arabia; msiddiquei@KSU.EDU.SA (M.M.S.); abuasrar@ksu.edu.sa (A.M.A.E.-A.); 4 Laboratory of Medical Biochemistry, University of Antwerp, 2610 Antwerp, Belgium; Pieter.VanWielendaele@uantwerpen.be (P.V.W.); ingrid.demeester@uantwerpen.be (I.D.M.)

**Keywords:** chemokine-derived peptide, heparan sulfate, growth factors, anti-angiogenic activity

## Abstract

**Simple Summary:**

Major angiogenic growth factors activate downstream signaling cascades by interacting with both receptor tyrosine kinases (RTKs) and cell surface proteoglycans, such as heparan sulfate proteoglycans (HSPGs). As current anti-angiogenesis regimens in cancer are often faced with resistance, alternative therapeutic strategies are highly needed. The aim of our study was to investigate the impact on angiogenic signaling when we interfered with growth factor-HSPG interactions using a CXCL9 chemokine-derived peptide with high affinity for HS.

**Abstract:**

Growth factors such as vascular endothelial growth factor (VEGF), fibroblast growth factor (FGF) and epidermal growth factor (EGF) are important angiogenesis-mediating factors. They exert their effects not only through their respective receptor tyrosine kinases (RTKs), but they also require molecular pairing with heparan sulfate proteoglycans (HSPGs). Angiogenic growth factors and their signaling pathways are commonly targeted in current anti-angiogenic cancer therapies but have unfortunately insufficient impact on patient survival. Considering their obvious role in pathological angiogenesis, HS-targeting drugs have become an appealing new strategy. Therefore, we aimed to reduce angiogenesis through interference with growth factor-HS binding and downstream signaling using a CXCL9-derived peptide with a high affinity for glycosaminoglycans (GAGs), CXCL9(74-103). We showed that CXCL9(74-103) reduced EGF-, VEGF165- and FGF-2-mediated angiogenic processes in vitro, such as endothelial cell proliferation, chemotaxis, adhesion and sprouting, without exerting cell toxicity. CXCL9(74-103) interfered with growth factor signaling in diverse ways, e.g., by diminishing VEGF165 binding to HS and by direct association with FGF-2. The dependency of CXCL9(74-103) on HS for binding to HMVECs and for exerting its anti-angiogenic activity was also demonstrated. In vivo, CXCL9(74-103) attenuated neovascularization in the Matrigel plug assay, the corneal cauterization assay and in MDA-MB-231 breast cancer xenografts. Additionally, CXCL9(74-103) reduced vascular leakage in the retina of diabetic rats. In contrast, CXCL9(86-103), a peptide with low GAG affinity, showed no overall anti-angiogenic activity. Altogether, our results indicate that CXCL9(74-103) reduces angiogenesis by interfering with multiple HS-dependent growth factor signaling pathways.

## 1. Introduction

Angiogenesis encompasses the formation of new blood vessels from an already mature vasculature. It comprises endothelial cell sprouting from the vessel wall in response to angiogenic stimuli, followed by degradation of the surrounding extracellular matrix (ECM) and migration through poorly-perfused tissues to extend the existing endothelial network. In physiological conditions, this is a well-orchestrated and controlled process balanced by pro- versus anti-angiogenic signals. On the other hand, in pathological angiogenesis, the tight control and balance is lost. This leads to immature, leaky vessels as observed in cancer and is a prerequisite not only for persistent growth of the tumor mass, but also for distant metastasis. Some of the angiogenic signals modulating neo-angiogenesis include vascular endothelial growth factor-A (VEGF-A), basic fibroblast growth factor (FGF-2) and epidermal growth factor (EGF) that bind with high specificity to their respective receptor tyrosine kinases (RTKs) and initiate downstream signaling cascades [1,2,3,4].

Over the years, it has become evident that these angiogenic signaling events not only require RTKs, but also involve active participation of proteoglycans [5]. Proteoglycans (PGs) consist of a protein core to which long, linear, negatively charged heteropolysaccharides, known as glycosaminoglycans (GAGs), are covalently linked. Based on the polysaccharide composition and sulfation pattern, GAGs are subdivided into four major families: heparin/heparan sulfate (Hep/HS), chondroitin/dermatan sulfate (CS/DS), keratan sulfate (KS) and hyaluronan (HA) [6]. This molecular superfamily is characterized by a high structural and spatial diversity, which makes its members highly promiscuous in their interactions and allows them to modulate a plethora of molecular processes [7,8]. Over the years, the role and importance of heparan sulfate proteoglycans (HSPGs) in angiogenesis and tumor progression has become clearer and efforts have been made to better delineate the specific processes in which they are involved [9,10]. Cell surface HSPGs aid in growth factor signaling by, e.g., interacting with both the growth factor (GF) and its receptor (GFR), which is reported for VEGF and FGF. As such, they facilitate the formation of a stable HS:GF:GFR complex allowing more potent and prolonged downstream signaling initiating differentiation, proliferation, migration, cell-cell and cell-matrix adhesion [11,12,13,14]. In addition, HSPGs have also been reported to facilitate signaling cascades via integrins, which adds to the complexity of their interactome [15]. EGF itself is not classified as a heparin-binding growth factor, but its receptor, EGFR, was shown to associate with the cell surface HSPG, syndecan-4, and integrins α_6_β_4_ and α_3_β_1_ on epithelial cells. This signaling complex mediated EGF-induced cell motility [16]. Genetic studies have underlined the importance of HS in signaling networks of several heparin-binding pro-tumoral growth factors such as VEGF, FGF, platelet-derived growth factor B (PDGF-B), transforming growth factor-beta (TGF-β), but also bone morphogenetic protein (BMP) and sonic hedgehog (Shh) [17]. For example, endothelial-specific targeting of HS in mice resulted in altered tumor angiogenesis with smaller tumors and vascular networks with reduced density and branching [18]. As a consequence of reduced HS production, FGF-2 and VEGF binding to mutant endothelial cells propagated in vitro was also dramatically reduced and exhibited reduced sprouting and extracellular signal-regulated kinase (ERK) phosphorylation [18]. 

Since angiogenesis takes a leading role in the progression of cancer, targeting its molecular players has been an area of intense investigation. Current anti-angiogenic strategies in cancer mainly target RTKs and sequestration of the growth factor itself, however, with limited success in terms of survival in cancer patients due to the acquired resistance and insufficient efficacy of therapy [19,20]. This emphasizes the need for other agents that target the vasculature [21]. Considering the obvious role of HS in tumor pathophysiology, HS-targeting drugs have become an appealing new strategy [10].

Our laboratory recently discovered that the COOH-terminal tail of the chemokine CXCL9, which is unusually long compared to most chemokines, is highly hydrophilic and has a high affinity for GAGs [22,23,24]. A COOH-terminal fragment comprising 30 amino acids, CXCL9(74-103), was considered the most potent GAG binder. This chemokine-derived peptide was characterized by a loss of CXCR3 receptor binding and signaling capacities. It could however effectively displace chemokines from GAGs, leading to a reduced chemokine-mediated neutrophil recruitment in various models of inflammation in vivo [22,23,24,25]. To further explore the applicability of CXCL9(74-103), and in light of the potential to target HS to reduce angiogenesis, we decided to investigate the anti-angiogenic properties of this high affinity GAG-binding peptide. We hypothesized that CXCL9(74-103) would interrupt the binding of angiogenic growth factors and/or their receptors to HSPGs and reduce or prevent the activation of their downstream signaling cascades.

Therefore, we first examined the effect of CXCL9(74-103) in a number of angiogenic growth factor- (FGF-2, VEGF165, EGF) mediated tumorigenic processes such as in vitro endothelial cell proliferation, migration and spheroid sprouting. As a negative control, we included a shorter CXCL9 COOH-terminal fragment, CXCL9(86-103), lacking 12 amino acids important for GAG binding. As a result, this CXCL9-derived peptide has a rather low affinity for GAGs. We studied the molecular mechanism of action through ELISA-like GAG and growth factor-binding assays and grating-coupled interferometry experiments. We then evaluated the anti-angiogenic potential of CXCL9(74-103) in various in vivo models, including the Matrigel plug assay, the corneal cauterization assay and an MDA-MB-231 breast cancer xenograft model.

## 2. Materials and Methods

### 2.1. Cell Culture and Reagents

Human microvascular endothelial cells (HMVECs) (Cell Systems, Kirkland, WA, USA) were cultured in endothelial cell basal medium-2 (EBM™-2) supplemented with the EGM™-2 MV SingleQuots kit (both Lonza, Basel, Switzerland) in an atmosphere of 5% CO_2_ at 37 °C. Mouse endothelial cells (MECs) were cultured in Dulbecco’s modified eagle medium (DMEM) (Gibco, Thermo Fisher Scientific, Waltham, MA, USA) supplemented with 20 mM HEPES, 1 mM sodium pyruvate and 10% (*v/v*) fetal calf serum (FCS) in 5% CO_2_ at 37 °C. Growth factors used were human recombinant VEGF165 (Biolegend, San Diego, CA, USA), human recombinant FGF-2 and EGF (both R&D Systems, Minneapolis, MN, USA) and murine recombinant VEGF165 (PeproTech, Cranbury, NJ, USA). When VEGF165 is mentioned as a stimulus, human VEGF165 is implied, unless specified otherwise. CXCL9 COOH-terminal peptides CXCL9(86-103) and CXCL9(74-103) were chemically synthesized using 9-fluorenyl methoxycarbonyl (Fmoc) solid-phase synthesis on an Activo-P11 automated peptide synthesizer (Activotec, Cambridge, UK), as previously described [26]. CXCL9(74-103) was site-specifically biotinylated or fluorescently labeled at the NH_2_-terminus using biotin-p-nitrophenyl ester (Novabiochem, Darmstadt, Germany), or 5(6)-carboxytetramethylrhodamine (TAMRA; Merck Millipore, Darmstadt, Germany), respectively, as described earlier [23,26]. The CXCL9 chemokine-based peptide sequences were KKVLKVRKSQRSRQKKTT and KKKQKNGKKHQKKKVLKVRKSQRSRQKKTT for CXCL9(86-103) and CXCL9(74-103), respectively.

### 2.2. Proliferation Assay 

The ability of the CXCL9-derived peptides to inhibit growth factor-induced proliferation of endothelial cells was assessed. HMVECs were seeded at 5 × 10^3^ cells/well in EBM™-2 medium containing 1% (*v/v*) FCS (proliferation medium). After settling of the cells, they were stimulated with EGF, VEGF165 (both 5 ng/mL) or FGF-2 (3 ng/mL) as a single stimulus or in combination with CXCL9(86-103) or CXCL9(74-103) at 0.3 or 3 µM in proliferation medium or left untreated. HMVECs were also treated with 0.3 or 3 µM of CXCL9(74-103) or 3 µM of CXCL9(86-103) alone to assess the effect of CXCL9-derived peptide treatment on spontaneous proliferation. After 3 to 4 days, a 3-(4,5-dimethylthiazol-2-yl)-2,5-diphenyl tetrazolium bromide (MTT) or ATP lite assay was performed. The ATP lite assay was performed according to the manufacturer’s instructions (Perkin Elmer, Waltham, MA). When MTT was used as substrate, the medium was replaced with 200 µL of MTT solution (RPMI without phenol red containing 0.4 g/L MTT) and the cells were incubated at 37 °C and 5% CO_2_ in the dark for 4 hours. Then, the MTT solution was discarded and the formed formazan crystals were dissolved in 200 µL acidic propanol [0.04 M HCl, 0.1% (*v/v*) NP-40 in isopropanol] under continuous shaking in the dark for 10 min. The optical densities were measured at 570 nm and 630 nm (reference wavelength).

### 2.3. xCELLigence Chemotaxis Assay

To evaluate the effect of the CXCL9-derived peptides on growth factor-induced endothelial cell migration, the xCELLigence^®^ real-time cell analyzer (RTCA DP) system (ACEA Biosciences, Inc.; San Diego, CA, USA) was used. First, 160 µL MCDB131 medium (Gibco) supplemented with 0.4% (*v/v*) FCS (control medium) or 160 µL control medium with 30 ng/mL FGF-2, 10 ng/mL VEGF165 or 10 ng/mL EGF were added to the lower chamber of a cell invasion/migration (CIM)-Plate (ACEA Biosciences, Inc.). After assembly of the lower and upper chamber, 50 µL of serum-free MCDB131 medium was added in the upper wells. Following equilibration of the plate at 37 °C for 1 h, HMVECs were added in the upper chamber at 4 × 10^4^ cells in 100 µL/well. CXCL9(74-103) or CXCL9(86-103) was added together with the cells in the upper compartment at a concentration of 0.3 or 3 µM. The assembled CIM-Plate was left at room temperature for 30 min to allow the cells to settle onto the membrane. Finally, the plate was placed in the instrument at 37 °C to monitor cell migration for 15 h. Cell migration from one compartment to the other was recorded (one recording every minute) as changes in electrical impedance. These changes were converted into cell indices, as a measure of cell migration.

### 2.4. xCELLigence Adhesion Assay

The wells of an E-plate (ACEA Biosciences, Inc.) were washed and coated with 0.1% (*v/v*) gelatin in phosphate-buffered saline (PBS) at 37 °C for 1 h. Following a washing step, wells were incubated with 0.1% (*v/v*) bovine serum albumin (BSA) in PBS at 37 °C for 1 h. After washing, 50 µL basal MCDB131 medium was added and the plate was allowed to equilibrate at 37 °C. HMVECs were harvested and seeded at 4 × 10^4^ cells/well together with 50 ng/mL VEGF165 with or without 0.03, 0.3 or 3 µM of CXCL9(74-103) or 3 µM of CXCL9(86-103) in 100 µL control medium (vide supra). Real-time changes in electrical impedance depicted as cell indices, as measure for cell adhesion and spreading, were analyzed using the xCELLigence^®^ RTCA DP System. The adhesion of HMVECs was analyzed 1 h after seeding.

### 2.5. Spheroid Sprouting Assay

A 1:5 mixture of endothelial cells (4 × 10^4^ cells/mL) and methylcellulose (Sigma-Aldrich, Saint Louis, MO, USA; 12 mg/mL in EBM™-2 basal medium) in EBM™-2 cell culture medium was prepared. Drops of 1 × 10^3^ HMVECs were plated on a petri dish and allowed to form single spheroids in hanging droplets at 37 °C, 5% CO_2_ for 24 h. Spheroids were collected, sedimented by centrifugation and resuspended in a mixture of 24 mg/mL methylcellulose in basal EBM™-2, 1 mg/mL collagen type I (BD Biosciences, San Jose, CA, USA), 2.5 mg/mL NaHCO_3_ and 10 mM NaOH. The suspension was plated in a 96-well plate and the collagen was allowed to polymerize at 37 °C, 5% CO_2_ for 30 min. Spheroids were left untreated or stimulated with 0.3 or 3 µM of CXCL9(74-103) or 3 µM of CXCL9(86-103) for 15 min prior to addition of 10 ng/mL FGF-2 or 10 ng/mL EGF in EBM™-2 + 3% (*v/v*) FCS. In some experiments, HMVECs were pretreated with heparinase II (vide infra). After 16 hours, spheroid sprouting was assessed with an inverted Axiovert 200M microscope (Zeiss, Germany) through a 10× objective. The average number of sprouts was calculated and the average sprout length per spheroid was measured using Fiji software.

### 2.6. In Vitro Toxicity Assay

HMVECs were seeded in MCDB131 medium supplemented with 3% (*v/v*) FCS at 8 × 10^3^ cells/well in a black, clear bottom 96-well plate coated with 0.1% (*v/v*) gelatin in PBS. The next day, cells were washed and incubated with control medium (vide supra) alone or in the presence of 0.3 or 3 µM of CXCL9(74-103) or CXCL9(86-103) for 24 h. The negative control cells were treated with 2% (*v/v*) Triton™ X-100 to induce cell death 21 h after the start of the experiment, i.e., 1 h prior to the addition of the LIVE/DEAD stain. The toxicity of the peptides was assessed using the LIVE/DEAD viability/cytotoxicity kit (Invitrogen, Carlsbad, CA, USA), according to the manufacturer’s instructions. At 24 h, calcein-acetoxymethyl ester (AM) and ethidium homodimer-1 (EthD-1) in 0.4% (*v/v*) FCS in DMEM FluoroBrite (Gibco) were added to all wells and the fluorescence was measured in the IncuCyte S3 live cell imaging system (Essen BioScience, Ltd.; Newark, UK).

### 2.7. Signal Transduction Assays

HMVECs and MECs were seeded in 6-well plates at 1.25 × 10^5^ cells/well in their respective culture medium. Once the cell density reached 70–80% confluency, the cells were starved in serum-free medium overnight. Fifteen minutes prior to stimulation, cells were incubated with various concentrations of CXCL9(74-103) or CXCL9(86-103) in 0.5% (*v/v*) BSA in MCDB131 or DMEM medium (assay medium for HMVECs and MECs, respectively) at 37 °C. HMVECs and MECs were stimulated with assay medium alone or supplemented with 10 ng/mL EGF or 5 ng/mL murine VEGF165 at 37 °C for 15 or 5 min, respectively. Then, the cells were placed on ice, washed with ice-cold PBS and 90 µL of lysis buffer [1% (*v*/*v*) protease inhibitor cocktail, phosphatase inhibitor cocktail 2 and 3 (all Sigma-Aldrich) in 1 mM ethylenediaminetetraacetic acid (EDTA), 0.5% (*v/v*) Triton™ X-100, 5 mM NaF, 6 M urea in PBS] was added per well. After a 15-minute incubation on ice, cells were collected using a cell scraper, transferred to pre-cooled tubes and incubated on ice for another 5 min. The cell lysates were centrifuged at 558 g and 4 °C for 5 min and the samples were stored at −20 °C until analysis. The amount of phospho-ERK1/2 in the cell lysates was determined using an ERK1 (Thr202/Tyr204)/ERK2 (Thr185/Tyr187) ELISA duoset (R&D Systems) according to the manufacturer’s instructions. The amount of phosphorylated ERK was normalized to the total protein content in the samples measured by the Pierce™ bicinchoninic acid (BCA) protein assay kit (Thermo Fisher Scientific).

### 2.8. ELISA-Like GAG Binding Assay 

The ability of the CXCL9-derived peptides to compete with VEGF165 for HS binding was assessed in an ELISA-like GAG binding assay. First, the GAG binding plates (Galen Laboratory Supplies, North Haven, CT, USA) were coated with 25 µg/mL HS (Iduron, Alderley Edge, UK) in standard assay buffer (SAB) [100 mM NaCl, 50 mM sodium acetate, 0.2% (*v/v*) Tween^®^ 20 in ultrapure water, pH 7.2] at room temperature overnight. The plates were washed with SAB and blocked with blocking buffer [SAB supplemented with 0.2% (*w/v*) gelatin] at 37 °C for 1 h to prevent nonspecific binding. Then, dilutions of VEGF165 and the peptides, CXCL9(74-103) or CXCL9(86-103), were added and allowed to interact at 37 °C for 2 h. Unbound material was removed through washing and biotinylated polyclonal goat anti-human VEGF165 antibody (PeproTech) in blocking buffer was added to interact with HS-bound VEGF165 at 37 °C for 1 h. Following additional washing steps, detection was performed with streptavidin-horseradish peroxidase (HRP) (R&D Systems) in blocking buffer at 37 °C for 30 min and conversion of 3,3’,5,5’-tetramethylbenzidine (TMB) to a colorimetric signal in the presence of 0.015% (*v/v*) H_2_O_2_. The reaction was stopped by the addition of H_2_SO_4_ and the absorbance was measured at 450 nm. All incubation steps were performed in the dark.

### 2.9. ELISA-Like Direct Binding Assay 

Direct interaction of CXCL9(74-103) and growth factors was assessed in an ELISA-like direct binding assay. The 96-well plates were coated with 50 ng/mL VEGF165, FGF-2 or EGF in PBS at 4 °C overnight. After washing with 0.05% (*v/v*) Tween^®^ 20 in PBS (washing buffer), the plate was incubated with washing buffer supplemented with 0.1% (*w/v*) casein (blocking buffer) at 37 °C for 1 h to prevent nonspecific binding. Dilutions of biotinylated CXCL9(74-103) in blocking buffer (1–1000 ng/mL) were added and allowed to interact at 37 °C for 1 h. Following additional washing steps, detection of the bound peptide was accomplished with streptavidin-HRP, as described above.

### 2.10. Grating-Coupled Interferometry (GCI)

GCI experiments were performed on a Creoptix WAVEdelta system (Creoptix AG, Wädenswil, Switzerland), which is a label-free surface biosensor system for the characterization of molecular interactions. 4PCP-STA chips (Creoptix AG) were used, which contain a quasi-planar polycarboxyl-matrix with pre-immobilized streptavidin. The running buffer for the experiments was PBS supplemented with 30 mM NaCl, 15 mM L-Arginine, 0.015% (*v/v*) Tween^®^ 20 and 0.05 mg/mL BSA at pH 7.4 for reducing nonspecific binding. Chips were conditioned using borate buffer (100 mM sodium borate, 1 M NaCl, pH 9.0). Capture was performed by injecting biotinylated CXCL9(74-103) at a concentration of 2 µg/mL and a flow rate of 10 µL/minute over the chip surface, until a density of +/− 160 was reached. After capture of biotinylated CXCL9(74-103), binding with FGF-2 was studied by injecting an FGF-2 concentration series over the chip surface. Since FGF-2 showed nonspecific binding on the surface, biotinylated antibodies (biotinylated polyclonal goat anti-rabbit immunoglobulins; Dako, Agilent, Santa Clara, CA, USA) were injected at a concentration of 10 µg/mL for 60 seconds for blocking the surface. A reference channel was blocked in the same way. Injections of the FGF-2 analyte were performed at 25 °C, with an association and dissociation time of 120 seconds and a flow rate of 40 µL/min. Absence of mass-transport limitation was verified by repeating the experiment at 50 µL/minute. A 1:3 dilution series with six dilutions (4.53 nM, 13.6 nM, 40.7 nM, 122 nM, 367 nM, 1.10 µM; all in duplicate) was analyzed. After each FGF-2 injection, a regeneration step was included, consisting of an injection of 2.5 M NaCl (40 µL/min flow rate, 30 sec injection time and 120 sec rinsing), followed by three injections of running buffer (same parameters as NaCl injection). Blank injections for referencing were included every 3rd sample cycle. Data adjustment (X and Y offset, dimethyl sulfoxide calibration and double referencing) and analysis (1:1 binding model with bulk correction) were performed using the Creoptix WAVEcontrol software.

### 2.11. Analysis of Cellular HS Binding of CXCL9(74-103)

CXCL9(74-103) binding to cellular HS was assessed via flow cytometry. HMVECs were either left untreated or incubated with 0.75 U/mL heparinase II (Sigma-Aldrich) at 37 °C for 2 h. Cells were harvested and left at room temperature for 1 h to recuperate, they were then placed on ice and centrifuged. After centrifugation (300 g, 4 °C, 7 min) and resuspension in PBS, 1 × 10^5^ cells per condition were stained with 3, 0.6 or 0.12 nM TAMRA-labeled CXCL9(74-103). To verify downregulation of HS expression, cells were stained with mouse anti-HS Ab (clone F58-10E4, Cat No 370255-S; Amsbio, Abingdon, UK) and R-PE goat anti-mouse IgM Ab (Cat No 115-116-075; Jackson ImmunoResearch, Westgrove, PA, USA). Flow cytometric analysis was performed using the BD LSRFortessa™ X-20 flow cytometer and FlowJo software (both BD Biosciences). 

Sprouting experiments examining the dependency on HS binding for the inhibitory action of CXCL9(74-103) were performed as described above (vide supra). Heparinase II treatment (0.75 U/mL) of spheroids was carried out 4 hours before starting the experiment and dilutions of EGF (10 ng/mL) and CXCL9(74-103) (3 µM) were additionally supplemented with 0.75 U/mL heparinase II.

### 2.12. Analysis of Retinal Vascular Permeability in the Rat Streptozotocin-Induced Diabetes Model

Male Sprague-Dawley rats (210–240 g) were fasted overnight and injected intraperitoneally (i.p.) with streptozotocin (55 mg/kg body weight, dissolved in citrate buffer, pH 4.5) to induce diabetes. After 3 days, the animals were anesthetized, diabetes was confirmed and the vitreous of the right eye was injected with equimolar amounts of CXCL9(74-103) (30 µg), CXCL9(86-103) (18 µg) or PBS (5 µL/injection). Diabetes-induced breakdown of the blood-retinal barrier as a consequence of increased vascular permeability was evaluated in retinas two weeks after streptozotocin injection, as previously described [27]. Briefly, 30 min prior to sacrifice, fluorescein isothiocyanate (FITC)-conjugated dextran of 3-5 kDa (Sigma-Aldrich) was intravenously (i.v.) injected. After collection of a blood sample, rats were perfused with PBS. The retinas were excised, weighed and homogenized. The fluorescence in the retina lysates was measured using a SpectraMax^®^ Gemini™ XPS system. For normalization of the data, the following equation was used to express the obtained results:

Retinal FITC-dextran (µg)/retinal weight (g)

Plasma FITC-dextran concentration (µg/µL) × circulation time (h)

Rat experiments were approved by the Institutional Animal Care and Committee of the College of Pharmacy, King Saud University.

### 2.13. In Vivo Angiogenesis Matrigel Plug Assay 

Six-to-eight-week-old female C57BL/6 mice were anesthetized with a mixture of 2.5 mg/mL xylazine (V.M.D, Inovet, Arendonk, Belgium) and 37.5 mg/mL ketamine (Dechra, Northwich, UK) in PBS by i.p. injection. A vertical dorsal incision was made and an ALZET osmotic pump (model 1007D, DURECT Corporation, Cupertine, CA, USA) filled with 400 µg/100 µL CXCL9(74-103) or 100 µL PBS was subcutaneously (s.c.) implanted. The incision was closed with a surgical suture. Growth factor-reduced Matrigel (Corning Matrigel Growth Factor (GFR) Basement Membrane Matrix, Phenol Red-Free, LDEV-Free; Corning Life Sciences, Amsterdam, The Netherlands) was s.c. injected in the dorsal flank forming a firm plug (600 µL/plug). The Matrigel was either injected as such (control plugs) or supplemented with 300 µg CXCL9(74-103) and/or 180 ng FGF-2. Seven days later, the mice were sacrificed. Thirty minutes before sacrifice, the mice were i.v. injected with 200 µL (25 mg/kg) FITC-labeled 2000 kDa dextran (Sigma-Aldrich) to quantify vessel formation in the Matrigel plugs. Blood was collected through cardiac puncture into heparinized tubes containing 20 µL heparin (5.000 IE/mL, Leo Pharma, Ballerup, Denmark). The blood was centrifuged at 200 g and 4 °C for 10 min, whereafter the supernatant was centrifuged at 3000 g and 4 °C for 10 min. The collected plasma was stored away from light at 4 °C until analysis. The Matrigel plugs were removed and enzymatically and mechanically digested in 1.2 mL dispase (Corning Life Sciences) using an incubation at 37 °C for 1.5 h and the gentleMACS™ Dissociator (Miltenyi Biotec, Bergisch Gladbach, Germany). The digested Matrigel plugs were filtered through a 70 µm cell strainer, washed with 1.5 mL FACS buffer [2% (*v*/*v*) FCS + 2 mM EDTA in PBS] and centrifuged at 300 g and 4 °C for 7 min. The supernatant (SN) was centrifuged again at 300 g and 4 °C for 7 min and the SN was retained away from light at 4 °C until analysis. The pellet was resuspended in 1 mL FACS buffer, the cells were counted and used for flow cytometry. The antibodies used to identify endothelial cells were anti-mouse CD45-APC (clone 30-F11, eBioscience, Thermo Fisher Scientific), anti-mouse CD146-PE (clone ME-9F1) and anti-mouse Pan-endothelial Cell Antigen-BV711 (clone MECA-32) antibodies (both BD Biosciences). Mice experiments were approved by the Ethical Committee of KU Leuven (project number P199/2018).

### 2.14. Corneal Cauterization Angiogenesis Assay 

The corneal cauterization assay was performed, as previously described [28]. Eight-week-old female C57BL/6 mice were anesthetized with a mixture of xylazine (10 mg/kg) and ketamine (100 mg/kg). After application of a local anesthetic (Unicaïne, 0.4%) to the eye, the cornea was thermally cauterized using an ophthalmic cautery. One day after cauterization, drops of 10 µL of CXCL9(74-103) (100 µg/mL) or PBS were applied daily for 4 days. On day 5, the mice were sacrificed, and the corneas were removed. Fixation and blocking of whole-mounted corneas were performed in 70% (*v/v*) ethanol for 1 h and 3% (*w/v*) BSA in PBS for 1 h, respectively. To stain for blood vessels, the corneas were incubated with rat anti-mouse CD31 antibody (clone MEC13.3, BD Biosciences) overnight and AlexaFluor568 goat anti-rat secondary antibody (Cat No A-11077, Invitrogen) for 2 h. Corneas were flat mounted on microscope glasses overlaid with Prolong Gold antifade mounting medium and imaged using a Leica DMI6000 microscope (Leica Microsystems, Wetzlar, Germany). The cornea blood vessel area was quantified using Leica MM AF morphometric analysis software (Leica Microsystems) and expressed as the percentage of the total corneal area. Ethical approval for animal experiments was obtained from the Ethical Committee of KU Leuven (number LA1210604).

### 2.15. In Vivo MDA-MB-231 Breast Cancer Mouse Model 

Six-to-eight-week-old female SCID mice were s.c. injected with 6 × 10^6^ MDA-MB-231 breast tumor cells (American Type Culture Collection, Manassas, VA) in 200 µL PBS. Three days later, mice were anesthetized as described (vide supra), a dorsal incision was made and an ALZET osmotic pump (model 1002, DURECT Corporation) containing 800 µg/100 µL CXCL9(74-103) or CXCL9(86-103) or 100 µL PBS was s.c. implanted, which delivered a continuous dose over the course of two weeks. Three weeks after tumor cell injection, mice were sacrificed. Tumors were isolated, minced and added to 2 mL of tumor cell medium [TCM; 5% (*v*/*v*) FCS in RPMI 1640 + Glutamax (Gibco)]. After centrifugation (500 g, room temperature, 5 min), minced tumors were incubated at 37 °C for 30 min in 3 mL of digestion medium [2 mg/mL collagenase D, 0.1 mg/mL DNAse and 5 U/mL dispase (Corning Life Sciences) in TCM]. Subsequently, tumor fragments were further disrupted using a 1 mL syringe and 18G needle, and 2 mL of digestion medium was added for an additional incubation at 37 °C for 15 min. Finally, mechanical disruption was repeated, followed by centrifugation of the suspension (500 g, room temperature, 5 min). To stop the digestion, the pellet was placed on ice and resuspended in 1 mL 10 mM EDTA in PBS. Then, 2 mL of 2% (*v*/*v*) FCS in PBS was added and the samples were centrifuged at 500 g and 4 °C for 5 min. The pellet was resuspended in 1 mL ACK lysis buffer (Gibco) to remove red blood cells and the reaction was stopped after 3 min by adding 4 mL of 2% (*v*/*v*) FCS in PBS. To remove debris, the sample was run through a 70 µm cell strainer, which was washed with 5 mL of 2% (*v/v*) FCS in PBS. After centrifugation (500 g, 4 °C, 5 min), the pellet was resuspended in 500 µL of 2% (*v/v*) FCS in PBS and the cells were counted. To assess the anti-angiogenic potential of the CXCL9-derived peptides, the population of endothelial cells within the tumor samples was determined via flow cytometry (vide supra).

### 2.16. Statistical Analysis

The data are represented as mean ± SEM. Statistical analysis was carried out by first performing a Kruskal-Wallis test. Comparisons between groups were performed using a Mann–Whitney U test for nonpaired data or a Wilcoxon test for paired data.

## 3. Results

### 3.1. CXCL9(74-103) Reduces Growth Factor-Induced Endothelial Cell Proliferation, Migration, Adhesion and Spheroid Sprouting In Vitro

**Figure 1:**
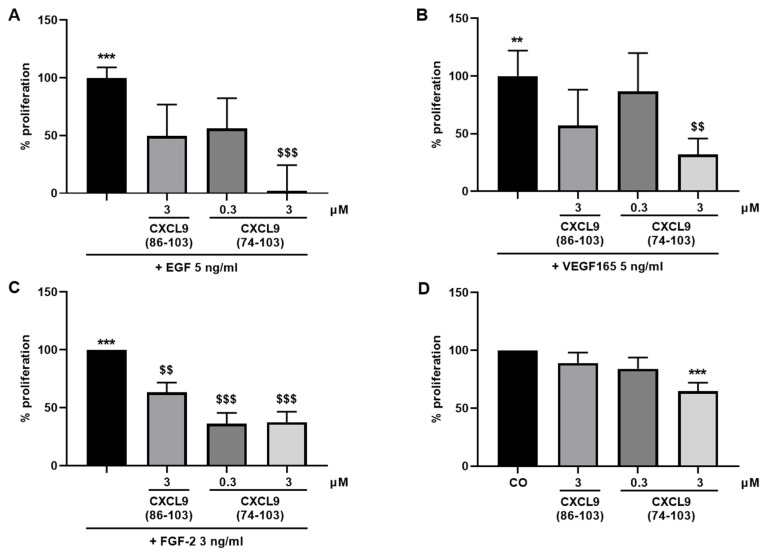
CXCL9(74-103) reduces growth factor-induced and basal endothelial cell proliferation. HMVECs were stimulated with (**A**) EGF, (**B**) VEGF165, or (**C**) FGF-2 as single stimulus or in combination with CXCL9(86-103) or CXCL9(74-103), or (**D**) CXCL9(86-103) and CXCL9(74-103) as a single stimulus at the indicated concentrations. After 3 to 4 days, the effect of the CXCL9-derived peptides on growth factor-induced and basal proliferation was determined. The mean (±SEM) proliferation compared to growth factor-induced (**A**–**C**) or compared to the control (**D**) (100%) is shown [n ≥ 4; Mann–Whitney U test; ** *p* < 0.01, *** *p* < 0.001 (compared to control); ^$$^ *p* < 0.01, ^$$$^ *p* < 0.001 (compared to growth factor)].

The ability of CXCL9 COOH-terminal peptides to interfere with different angiogenic processes was first evaluated in several in vitro assays. To start, the growth factor-induced proliferation of endothelial cells in the presence of the CXCL9-derived peptides was assessed. All three growth factors EGF, VEGF165 and FGF-2 induced a significant enhancement of proliferation of HMVECs, as expected. When endothelial cells were co-stimulated with CXCL9(74-103), endothelial cell proliferation was significantly reduced, in a dose-dependent manner (Figure 1). Treatment with 3 µM of CXCL9(74-103) almost completely abolished the proliferative effect of EGF (Figure 1A). Similarly, VEGF165- and FGF-2-induced proliferation were also significantly decreased when cells were treated with 3 µM of CXCL9(74-103) (Figure 1B,C). The lower dose (0.3 µM) of CXCL9(74-103), as well as the highest dose (3 µM) of CXCL9(86-103) could not significantly alter EGF- and VEGF165-induced proliferation. In contrast, treatment with 0.3 µM of CXCL9(74-103) and 3 µM of CXCL9(86-103) also significantly attenuated endothelial cell proliferation in response to FGF-2 (Figure 1C). Finally, basal HMVEC proliferation was also significantly reduced by CXCL9(74-103) at a dose of 3 µM (Figure 1D).

**Figure 2:**
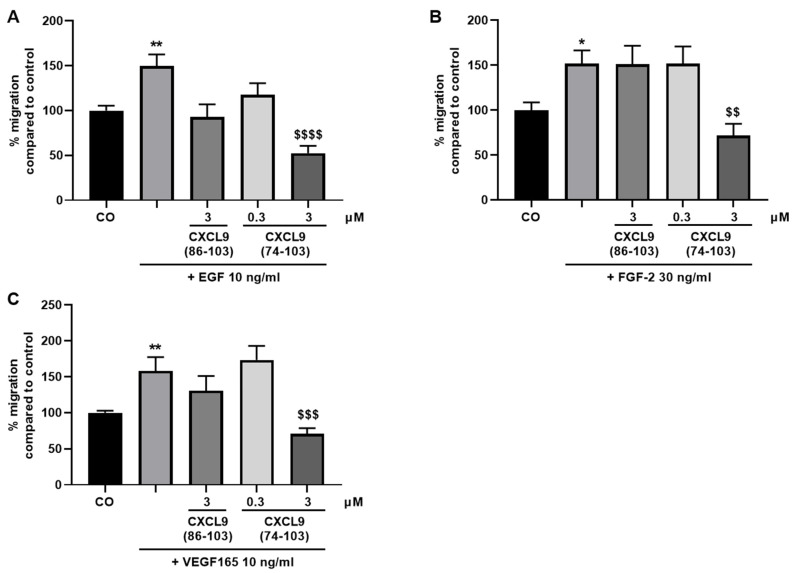
CXCL9(74-103) reduces growth factor-induced endothelial cell migration. HMVEC chemotaxis in a CIM plate towards (**A**) EGF, (**B**) FGF-2, or (**C**) VEGF165 in the presence or absence of CXCL9-derived peptides was measured using the xCELLigence RTCA DP System. Changes in electrical impedance were converted into cell indices as a measure of migration. The data are represented as a mean (±SEM) percentage of migration compared to the control (100%). [n ≥ 3; Mann–Whitney U test; * *p* < 0.05, ** *p* < 0.01 (compared to control); ^$$^ *p* < 0.01, ^$$$^ *p* < 0.001, ^$$$$^ *p* > 0.0001 (compared to growth factor)].

Next, endothelial cells were investigated for their chemotactic migration in response to angiogenic growth factors when treated with CXCL9(74-103) or CXCL9(86-103). The growth factors EGF, FGF-2 or VEGF165 were added in the lower chamber and the cells, together with CXCL9(74-103) or CXCL9(86-103), were added in the upper chamber. Migration of endothelial cells towards the growth factor in the lower chamber was measured using the RTCA DP system. EGF, FGF-2 and VEGF165 stimulation all caused a significant enhancement of endothelial cell migration to the lower chamber (Figure 2). CXCL9(86-103) at a dose of 3 µM was not able to counteract the growth factor-induced migration. CXCL9(74-103) at the same concentration of 3 µM could, however, significantly reduce the chemotactic migration of HMVECs towards EGF, FGF-2 and VEGF165 to baseline migration (cells treated with control medium alone).

**Figure 3:**
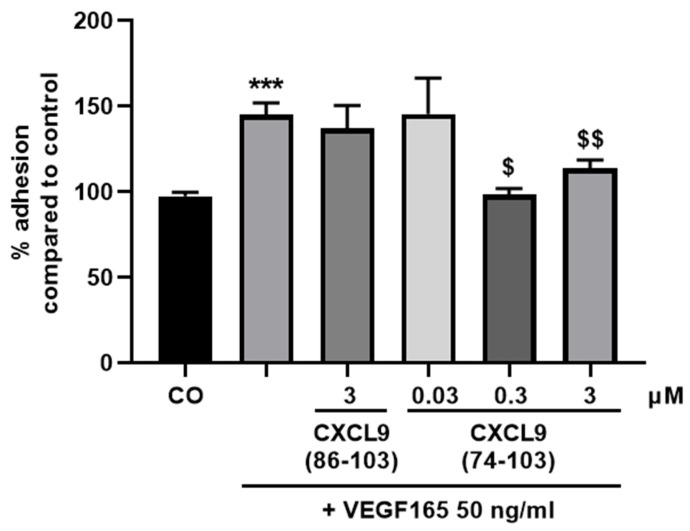
CXCL9(74-103) inhibits VEGF165-induced endothelial cell adhesion. HMVECs were added to gelatin-coated E-plates in the presence of 50 ng/mL VEGF165 alone or in combination with CXCL9(86-103) or CXCL9(74-103) at the indicated concentrations. The cell indices as a measure of adhesion were evaluated after 1 h. The results are expressed as a mean (±SEM) percentage adhesion compared to the control (100%) [*n* = 2–6; Mann–Whitney U test; *** *p* < 0.001 (compared to control); ^$^ *p* < 0.05, ^$$^ *p* < 0.01 (compared to VEGF165 50 ng/mL)].

We next assessed the influence of CXCL9(74-103) or CXCL9(86-103) treatment on the ability of endothelial cells to adhere to and spread out on a gelatin-coated surface in response to VEGF165. Endothelial cell adhesion was measured using gold microelectrode-coated E-plates and the RTCA DP system, as previously described [29]. In response to VEGF165, HMVECs adhered more profoundly to the substratum compared to untreated cells (Figure 3). No effect on VEGF-induced adhesion was seen by treating HMVECs with CXCL9(86-103). CXCL9(74-103) could completely revert the effect of VEGF165 on endothelial cell adhesion both at 0.3 and 3 µM.

**Figure 4:**
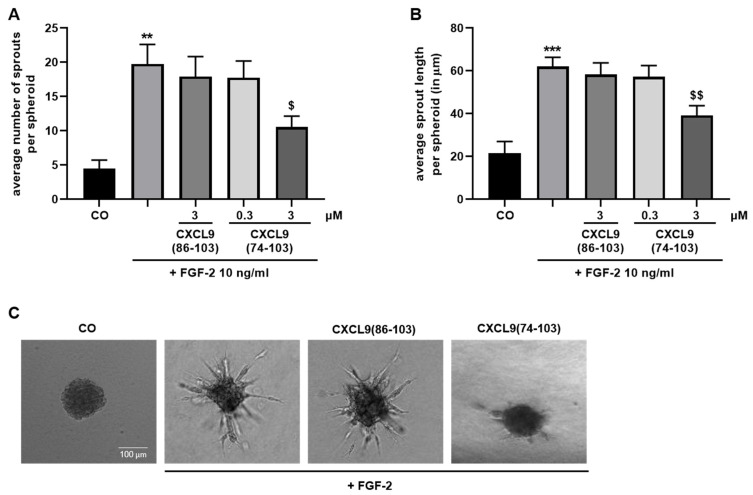
CXCL9(74-103) reduces FGF-2-mediated spheroid sprouting of endothelial cells. Collagen-embedded HMVEC spheroid sprouting in response to 10 ng/mL FGF-2 alone or with CXCL9(86-103) or CXCL9(74-103) at the indicated concentrations was assessed microscopically after 16 hours. The number of sprouts per spheroid was counted and the sprout length was measured using Fiji software. (**A**) Average [mean (±SEM)] number of sprouts per spheroid and (**B**) average sprout length per spheroid (in µm) are depicted. (**C**) Representative images of spheroids that were untreated (CO), stimulated with FGF-2 with or without 3 µM of the indicated CXCL9-derived peptide are shown. Scale bar = 100 µm. [*n* = 5 experiments, ≥3 spheroids per experiment; Mann–Whitney U test; ** *p* < 0.01, *** *p* < 0.001 (compared to control); ^$^ *p* < 0.05, ^$$^ *p* < 0.01 (compared to FGF-2)].

The spheroid sprouting assay is a robust in vitro angiogenesis model as it allows the study of angiogenesis in a 3D environment and shares prominent characteristics with the in vivo situation [30]. HMVEC spheroids embedded in a collagen matrix responded to FGF-2 treatment by pronounced endothelial sprouting after 16 hours (Figure 4). The number of sprouts, as well as the sprout length of FGF-2-treated spheroids, were diminished when spheroids were incubated with 3 µM of CXCL9(74-103). CXCL9(86-103) at 3 µM had no effect on sprout outgrowth induced by FGF-2. 

Altogether, these in vitro data show that CXCL9(74-103), but not CXCL9(86-103), can interfere with growth factor-induced processes including endothelial cell proliferation (VEGF, FGF-2, EGF), migration (VEGF, FGF-2, EGF), adhesion (VEGF) and spheroid sprouting (FGF-2). 

### 3.2. CXCL9 COOH-Terminal Peptides Are Not Toxic to Primary Endothelial Cells

**Figure 5:**
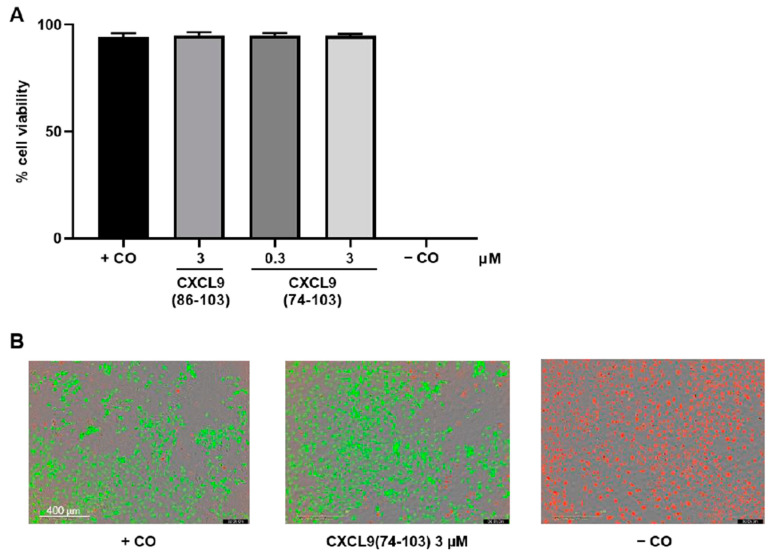
CXCL9-derived peptides are not toxic to primary endothelial cells. HMVECs were treated with CXCL9-derived peptides (0, 0.3 or 3 µM). (**A**) After 24 h, the toxicity was assessed using the LIVE/DEAD viability/cytotoxicity assay. The fluorescence was measured using the IncuCyte S3 live cell imaging system. Viable cells were stained with calcein-AM (green) and dead cells with ethidium homodimer-1 (red). Positive control (+CO) cells were untreated. Negative control (−CO) cells were incubated with 2% (*v*/*v*) Triton X-100 to induce cell death. The mean (±SEM) percentage of cell viability is depicted (*n* = 4). (**B**) Representative images of positive and negative control cells and cells treated with 3 µM CXCL9(74-103) are shown. Scale bar = 400 µm.

To ascertain that the in vitro effects of the CXCL9-derived peptides were not due to any induced toxicity, a viability/cytotoxicity assay was performed. This assay uses two fluorescent dyes, calcein-AM, a marker for viable cells and EthD-1, a marker for cell death. The fluorescence was measured using the IncuCyte S3 live cell imaging system. After seeding HMVECs in a medium with low serum percentage, cells were incubated with 0.3 µM or 3 µM of the CXCL9-derived peptides for 24 h. Cells that served as negative control were incubated with 2% (*v/v*) Triton™ X-100 for 1 h before the end of the 24-h incubation to induce cell death (Figure 5B, panel far right). No significant differences in cell viability between cells treated with or without CXCL9-derived peptides were observed (Figure 5A,B), indicating that the peptides are not toxic to primary endothelial cells.

### 3.3. CXCL9(74-103) Interferes with EGF- and VEGF165-Induced ERK Phosphorylation

**Figure 6:**
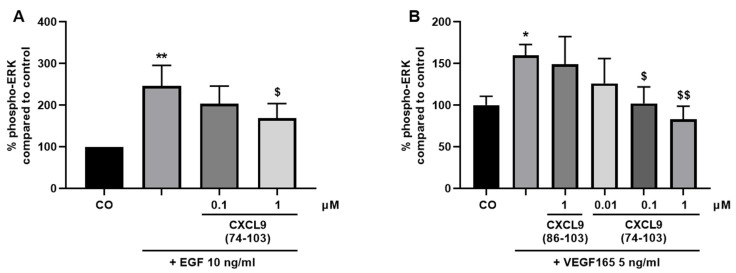
CXCL9(74-103) reduces EGF- and VEGF165-induced activation of pERK in endothelial cells. (**A**) HMVECs or (**B**) MECs were stimulated with (**A**) 10 ng/mL EGF or (**B**) 5 ng/mL murine VEGF165 as a single stimulus or with varying concentrations of CXCL9(86-103) or CXCL9(74-103) for (**A**) 15, or (**B**) 5 min, respectively. The amount of phosphorylated ERK to total protein was determined. Results are expressed as a mean (±SEM) percentage of phospho-ERK compared to the control (100%). [n ≥ 3; Mann–Whitney U test; * *p* < 0.05, ** *p* < 0.01 (compared to control); ^$^ *p* < 0.05, ^$$^ *p* < 0.01 (compared to growth factor)].

Growth factor binding to their respective RTKs can induce phosphorylation of ERK, which induces proliferation and cell growth. To further examine how CXCL9(74-103) targets growth factor-mediated processes, this signal transduction pathway was investigated. First, HMVECs or MECs were treated for 15 min with CXCL9(86-103) or CXCL9(74-103). Then, HMVECs were stimulated with 10 ng/mL EGF for 15 min and MECs with 5 ng/mL murine VEGF165 for 5 min, to induce phosphorylation of ERK. Stimulation of HMVECs with EGF enhanced the phospho-ERK content to 247% (Figure 6A). This increase was significantly attenuated by CXCL9(74-103) at 1 µM. The upregulation of phosphorylated ERK in response to VEGF165 was also significantly decreased in the presence of 0.1 or 1 µM of CXCL9(74-103), from 160% to baseline levels (Figure 6B). Treatment with CXCL9(86-103) at 1 µM did not result in a reduction of VEGF165-induced ERK phosphorylation. 

### 3.4. CXCL9(74-103) Competes with VEGF165 for GAG Binding and Directly Interacts with FGF-2

**Figure 7:**
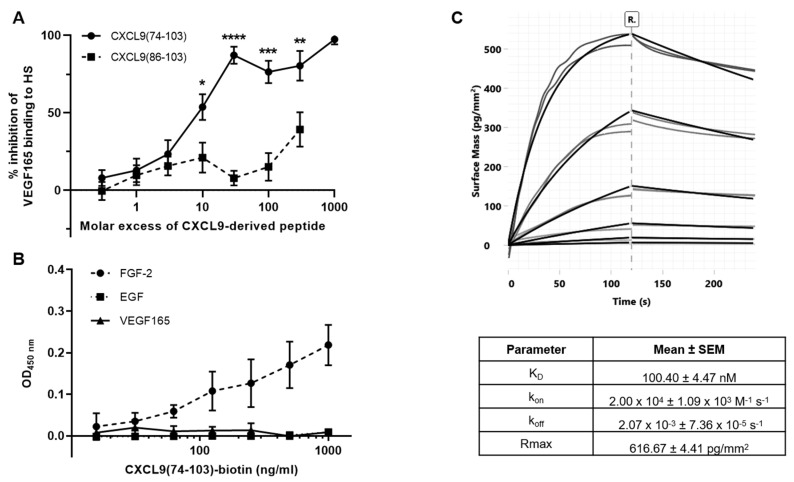
CXCL9(74-103) competes with VEGF165 for HS binding and directly interacts with FGF-2. (**A**) Competition between CXCL9(86-103) and CXCL9(74-103) with VEGF165 for HS binding was examined in an ELISA-like GAG binding assay. Results are expressed as a mean (±SEM) percentage inhibition of VEGF165 binding to HS per indicated molar excess of CXCL9-derived peptide. [*n* = 5; Mann–Whitney U test; * *p* < 0.05, ** *p* < 0.01, *** *p* < 0.001, **** *p* < 0.0001 [compared to CXCL9(86-103)]]. (**B**) Direct interaction of CXCL9(74-103) with FGF-2, EGF or VEGF165 was determined. Plates were coated with 50 ng/mL growth factor and interaction with CXCL9(74-103)-biotin was detected with streptavidin-HRP. The mean (±SEM) OD value measured at a wavelength of 450 nm for each indicated concentration of CXCL9(74-103)-biotin is shown (n ≥ 2, in duplicate). (**C**) Direct interaction between FGF-2 and CXCL9(74-103) was characterized using grating-coupled interferometry. A representative sensorgram (grey: experimental data; black: data fitting) and a table with the mean kinetic parameters (±SEM) are shown (*n* = 3). The sensorgram shows binding of a concentration series of FGF-2 (4.53 nM, 13.6 nM, 40.7 nM, 122 nM, 367 nM, 1.10 µM; in duplicate) flown over biotinylated CXCL9(74-103) immobilized on a streptavidin-coated sensorchip. The data are represented as the mass bound to the chip surface (pg/mm^2^) in function of time (s), using double referencing against a reference channel and blank buffer injections.

To further unravel the molecular mechanisms that play part in the observed inhibitory effects of CXCL9(74-103), both CXCL9 COOH-terminal peptides were tested for their ability to compete with VEGF165 for HS binding in an ELISA-like GAG binding assay. CXCL9(74-103) efficiently competed with VEGF165 for binding to HS already at a 10-fold molar excess of peptide attaining 50% inhibition (Figure 7A). At 30-fold molar excess of CXCL9(74-103) over VEGF165, an inhibition percentage of 90% was reached. This is in contrast to CXCL9(86-103), that only showed ≈20% inhibition at a 30-fold excess and 40% inhibition only at a 300-fold excess. Significant differences in inhibition were observed between CXCL9(74-103) and CXCL9(86-103) already from a 10-fold molar excess of CXCL9-derived peptide. As not EGF, but its receptor EGFR, directly interacts with HS, competition for HS binding between EGF and CXCL9(74-103) was not evaluated. We could not detect any clear competition between FGF-2 and CXCL9(74-103) for HS binding (data not shown). However, we found that FGF-2 was the only one of the growth factors that directly interacted with CXCL9(74-103) in an ELISA-like direct binding assay (Figure 7B). VEGF165 and EGF did not interact with CXCL9(74-103). The direct interaction of FGF-2 with CXCL9(74-103) can explain why we could not observe competition for HS binding specifically between FGF-2 and CXCL9(74-103). We confirmed and further characterized the interaction of FGF-2 with CXCL9(74-103) using GCI analysis (Figure 7C). A concentration series of FGF-2 was injected in duplicate over a streptavidin-coated sensor chip with immobilized biotinylated CXCL9(74-103). After each FGF-2 injection, the immobilized biotinylated CXCL9(74-103) was regenerated using injections of 2.5 M NaCl. The ascending part of each binding curve represents the association phase (0–120 s), which is followed by the dissociation phase (120–240 s). Based on the on- and off-rate constants, k_on_ and k_off_, the binding affinity (K_D_) was determined. The obtained K_D_ of 100.4 nM further supported a direct association with moderate affinity between CXCL9(74-103) and FGF-2 (Figure 7C). 

### 3.5. CXCL9(74-103) Depends on HS for Binding and Its Mechanism of Action

**Figure 8:**
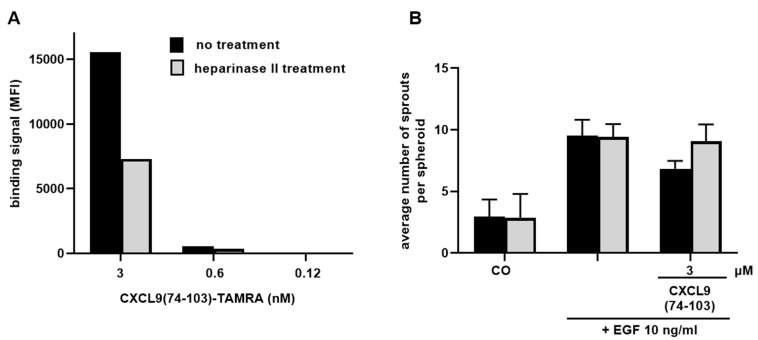
CXCL9(74-103) relies on cell surface HS for binding to endothelial cells and for exerting its anti-angiogenic activity. (**A**) The binding of different nanomolar concentrations of fluorescent TAMRA-labeled CXCL9(74-103) to HMVECs after incubation with or without 0.75 U/mL of heparinase II at 37 °C for 2 h was evaluated via flow cytometry. The fluorescence was quantified, and the median fluorescence intensity (MFI) is shown. Results of 1 representative experiment out of 3 independent experiments are shown. (**B**) The average number of sprouts per spheroid in response to 10 ng/mL EGF and the inhibition thereof in the presence of 3 µM CXCL9(74-103) was analyzed when spheroids were treated with heparinase II or left untreated. The results shown [mean (±SEM)] are derived from two independent experiments.

To ascertain that CXCL9(74-103) relies on HS for its anti-angiogenic activity, two types of experiments were set up. The efficiency of CXCL9(74-103) binding to HMVECs after treatment with heparinase II, an enzyme responsible for the digestion of HS, was first evaluated. Endothelial cells were either treated with heparinase II for 2 h or left untreated and the binding of different molar concentrations of fluorescent TAMRA-labeled CXCL9(74-103) was thereafter evaluated with flow cytometry. Untreated HMVECs bound CXCL9(74-103) in a concentration-dependent manner and heparinase II-treated endothelial cells bound CXCL9(74-103) less efficiently compared to cells with intact HS (Figure 8A). Downregulation of HS expression following heparinase treatment was verified in each experiment, which corresponded to a reduction of minimally 50% in HS fluorescence intensity (data not shown). Then, the dependency of CXCL9(74-103) on HS binding for its inhibitory action was evaluated. A spheroid sprouting experiment was carried out, wherein HMVEC spheroids were stimulated with 10 ng/mL EGF with or without 3 µM of CXCL9(74-103) after treatment with heparinase II. In both experiments, CXCL9(74-103) inhibited EGF-mediated spheroid sprouting in control conditions, which was not observed upon heparinase II treatment, demonstrating that HS is involved in the anti-angiogenic activity of CXCL9(74-103) (Figure 8B). In the case of EGF, it is not the growth factor ligand, but the receptor, EGFR, that relies on HS and integrin pairing for the formation of a signaling complex.

We continued our research by providing in vivo evidence that CXCL9(74-103) could inhibit angiogenesis induced by either VEGF165, FGF-2 or EGF. We used models wherein one of these growth factors is the dominant actor, as well as a model wherein several growth factors act in concert.

### 3.6. CXCL9(74-103) Inhibits In Vivo Diabetes-Induced Vascular Permeability

**Figure 9:**
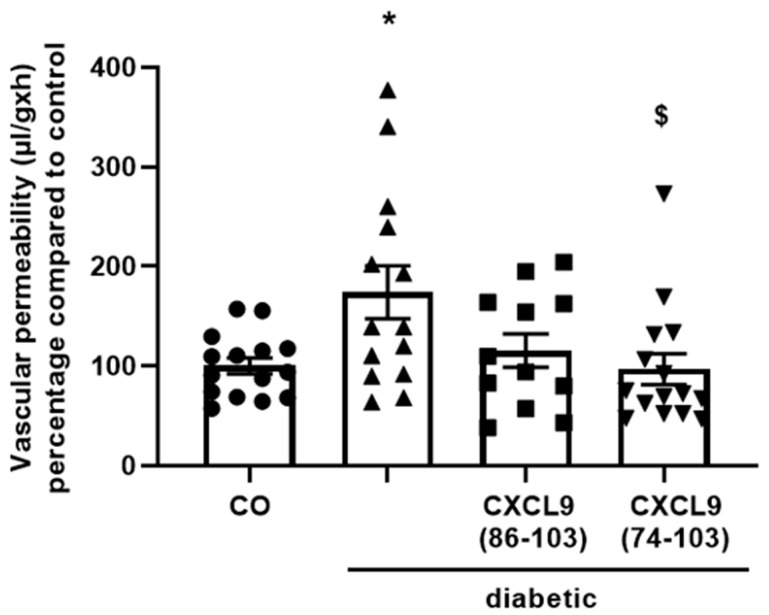
CXCL9(74-103) reduces vascular permeability in diabetes-induced rats. After induction of diabetes in rats, the vitreous of the right eye was injected with equimolar amounts of CXCL9(74-103) (30 µg), CXCL9(86-103) (18 µg) or PBS. After two weeks, the retinas were excised and the vascular leakage was assessed using FITC-labeled dextran i.v. injected prior to sacrifice. Vascular permeability (µL/g × h) [mean (±SEM)] is depicted compared to control rats. [Mann–Whitney U test; * *p* < 0.05 (compared to control rats); ^$^ *p* < 0.05 (compared to diabetic rats); CO rats: *n* = 15, diabetic rats: *n* = 14, CXCL9(86-103)-treated diabetic rats: *n* = 12, CXCL9(74-103)-treated diabetic rats: *n* = 15; the experiment was repeated three times].

VEGF is a major contributor to vascular permeability and neovascularization in diabetic retinopathy and tumor growth [20,31]. Although it has not yet been established whether VEGF-induced leakage in diabetic retinopathy is directly mediated by VEGF or by secondary mediators, the fact that patients benefit from anti-VEGF therapy underlines its principal role in blood-retinal barrier (BRB) breakdown [32]. Therefore, an in vivo diabetes-induced vascular permeability assay was included to assess interference of CXCL9(74-103) with this VEGF-induced process. After induction of diabetes with streptozotocin, the vitreous of the right eye was injected with equimolar amounts of CXCL9(74-103), CXCL9(86-103) or PBS as vehicle. After two weeks, vascular leakage was assessed by i.v. injection of FITC-labeled dextran prior to sacrifice and subsequent measurement of the fluorescence intensity in the retinal homogenates. Vascular permeability was significantly increased in diabetic rats compared to healthy rats (Figure 9). Treatment of diabetic rats with CXCL9(86-103) tended to decrease the vascular leakage, but this was not significant. When diabetic rats were treated with CXCL9(74-103), there was a significant improvement of the vascular leakage and recovery of the BRB to a level which was comparable to healthy PBS-treated rats. 

### 3.7. CXCL9(74-103) Reduces In Vivo Angiogenesis in the Matrigel Plug Assay, the Corneal Cauterization Assay and in Xenografts of Human Breast Carcinoma 

In the Matrigel plug assay, 180 ng FGF-2 was embedded in Matrigel together with 300 µg CXCL9(74-103). FGF-2 was used as the growth factor of choice in these experiments, as it was more potent than VEGF165 and EGF. Since Matrigel matrix is charged with HS, the plug was loaded with a high dose of CXCL9-derived peptide to saturate the available binding sites. In addition, as CXCL9 COOH-terminal peptides have a relatively short half-life, a continuous supply of the CXCL9-derived peptide (400 µg over 7 days) was accomplished by dorsal, s.c. implantation of an ALZET osmotic pump. As one measure for angiogenesis, high molecular weight FITC-dextran unable to diffuse from blood vessels, was injected i.v. 30 min before sacrifice. The ratio of fluorescence in the Matrigel plug to fluorescence in the plasma was used to quantify the amount of blood vessels formed in the plug. Another measure for angiogenesis comprised counting the number of cells inside the plugs and staining with antibodies against the endothelial cell markers CD146 and MECA-32 for flow cytometry.

**Figure 10:**
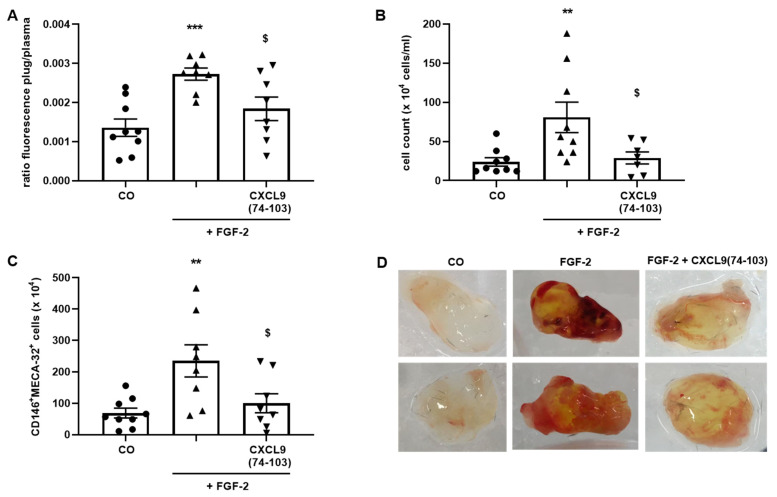
CXCL9(74-103) reduces FGF-2-induced neovascularization. Matrigel loaded with 180 ng FGF-2 with or without 300 µg CXCL9(74-103) was s.c. injected in mice. Dorsal s.c. implanted pumps allowed the gradual release of CXCL9(74-103) (400 µg over 7 days) or PBS. Seven days later, the mice were i.v. injected with high molecular weight FITC-dextran 30 min before sacrifice. The plugs were digested and (**A**) the ratio of fluorescence in the plug compared to plasma was determined, (**B**) the number of cells inside the plugs was counted and (**C**) the endothelial cell population was analyzed with flow cytometry. (**D**) Representative images of control, FGF-2 and FGF-2 + CXCL9(74-103) plugs are depicted. [mean (±SEM); Mann–Whitney U test; ** *p* < 0.01, *** *p* < 0.001 (compared to control plugs); ^$^ *p* < 0.05 (compared to FGF-2-containing plugs); (CO group: *n* = 9, FGF-2 group: *n* = 8–9, FGF-2 + CXCL9(74-103) group: *n* = 7–8); the experiment was repeated three times].

Addition of FGF-2 to the Matrigel plug doubled the fluorescence ratio compared to the control (Figure 10A). The number of cells inside the plugs and the CD146^+^MECA-32^+^ cell population almost increased 3-fold in FGF-2-treated plugs compared to control plugs (Figure 10B,C). Macroscopical observations confirmed these findings as FGF-2-treated plugs clearly appeared more perfused than control plugs (Figure 10D). CXCL9(74-103) was able to significantly reduce FGF-2-induced angiogenesis in the Matrigel plugs. The fluorescence ratio, total number of cells, as well as the number of CD146^+^MECA-32^+^ cells recruited by FGF-2 were decreased by CXCL9(74-103) (Figure 10A–C). Accordingly, FGF-2-containing Matrigel plugs of mice treated with CXCL9(74-103) were more transparent compared to FGF-2-containing plugs of untreated mice (Figure 10D). 

**Figure 11:**
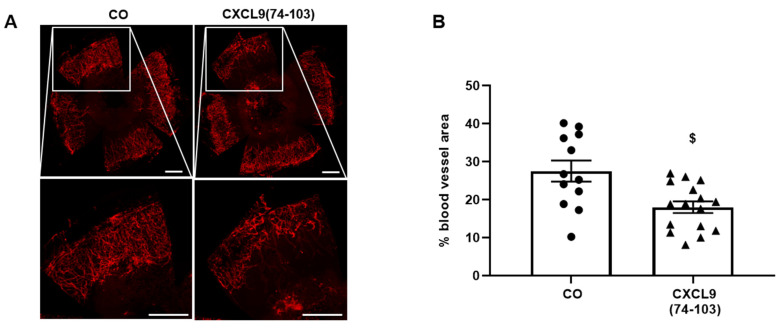
CXCL9(74-103) decreases pathological blood vessel outgrowth in response to thermal corneal injury. Mice were anesthetized and retinas were thermally cauterized on day 1. From day 2, the corneas were treated topically with drops of 100 µg/mL of CXCL9(74-103) or PBS for 4 days. On day 5 post-injury, corneas were excised and blood vessel outgrowth was visualized and quantified through fluorescent staining of CD31. (**A**) Representative images of thermal injury-induced corneal CD31^+^ blood vessels (red) and (**B**) quantification of CD31^+^ blood vessel area of PBS- and CXCL9(74-103)-treated corneas are shown. Scale bar = 500 µm. [mean (±SEM); Mann–Whitney U test; ^$^ *p* < 0.05 (compared to PBS-treated corneas); (CO group: *n* = 12, CXCL9(74-103)-treated group: *n* = 16); the experiment was repeated three times].

In the corneal cauterization assay, neovascularization in the cornea occurs to repair the damage inflicted by thermal cauterization. The most important player in this model of angiogenesis is VEGF-A, but also an upregulation of TGF-β and CCL2 have been reported [33,34,35]. One day after cauterization, mice corneas were treated daily by topical application of 100 µg/mL CXCL9(74-103) or PBS for 4 days. The induced blood vessel area was assessed on day 5 post-injury by isolation of the cornea and fluorescent staining of the endothelial cell marker CD31. Representative images clearly show a reduction in CD31^+^ blood vessel outgrowth in CXCL9(74-103)-treated corneas (Figure 11A). Quantification of the CD31^+^ area demonstrated a significant reduction of blood vessel area in corneas treated with ophthalmic drops of CXCL9(74-103) (Figure 11B).

**Figure 12:**
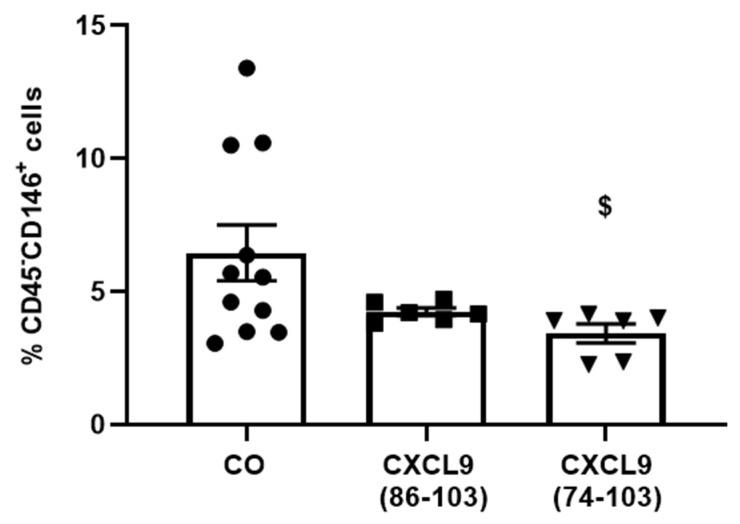
CXCL9(74-103) attenuates tumor angiogenesis in an EGF-dependent MDA-MB-231 breast carcinoma model. Three days after s.c. MDA-MB-231 tumor cell injection, osmotic pumps containing CXCL9(86-103), CXCL9(74-103) [800 µg in 100 µL] or PBS were s.c. implanted in tumor-bearing SCID mice. The osmotic pumps allowed the gradual release of treatment for two weeks. Three weeks after tumor cell injection, tumors were removed and digested, and the endothelial cell population within the tumors was analyzed through flow cytometry. [mean (±SEM); Mann–Whitney U test; ^$^ *p* < 0.05 (compared to PBS-treated tumor-bearing mice); (CO group: *n* = 11, CXCL9(86-103)-treated group: *n* = 6, CXCL9(74-103)-treated group: *n* = 6); the experiment was performed twice].

The anti-angiogenic effect of CXCL9(74-103) was further evaluated in an EGF-dependent breast cancer model. We made use of MDA-MB-231 cells, which are highly metastatic triple-negative breast cancer cells that express high levels of EGFR. This breast carcinoma model is therefore suitable to study the anti-angiogenic effect of CXCL9(74-103) in a tumor microenvironment highly dependent on EGFR signaling [36]. Three days after s.c. tumor cell injection, osmotic pumps containing 800 µg of CXCL9(74-103), 800 µg of CXCL9(86-103) or PBS were s.c. implanted. Three weeks after tumor cell injection, the endothelial cell population within the tumor was determined by flow cytometry. The percentage of CD45^−^CD146^+^ cells, identified as endothelial cells, was significantly reduced in tumors of CXCL9(74-103)-treated mice compared to tumors of PBS-treated mice (Figure 12). 

## 4. Discussion 

Glycosaminoglycans and their associated protein-bound form, proteoglycans, are implicated in numerous physiological and pathological processes. The importance of cell surface HSPGs, as integrators of growth factor signaling in angiogenesis has already been established in a number of studies [17]. In addition, deficiency in the major lymphatic HSPG, syndecan-4, was associated with a dysfunctional VEGF-C:VEGFR3 complex formation required for adequate VEGFR3 signaling and VEGF-C-mediated pathological lymphangiogenesis in vivo [37]. Given the importance of proteoglycans in pathological angiogenesis and the pressing need for additional angiogenesis inhibitors, targeting HS has become a field of intense investigation. In practice, however, the number of HS-targeting drugs is limited. 

In our present study, we wanted to explore the potential of CXCL9(74-103), a high affinity GAG-binding peptide, to interfere with angiogenesis by targeting growth factor-induced signaling cascades and associated processes. We found that CXCL9(74-103) efficiently inhibited multiple angiogenic factor-mediated processes in vitro such as proliferation (VEGF165, FGF-2, EGF), chemotaxis (VEGF165, FGF-2, EGF), adhesion (VEGF) and spheroid sprouting (FGF-2, EGF) of endothelial cells, without inducing any cell toxicity. These findings were corroborated by the observation that CXCL9(74-103) reduced neovascularization in several in vivo models, wherein one or more of the growth factors studied in vitro is the principal actor inducing angiogenesis. We opted for diabetic retinopathy for VEGF, the Matrigel plug assay for FGF-2 and a xenograft breast cancer model for EGF. In the corneal cauterization model, beside VEGF, TGF-β and CCL2 are also involved [33,34,35]. In our study, we made use of two CXCL9-derived COOH-terminal peptides. CXCL9(74-103) is characterized by a high affinity for GAGs, whereas CXCL9(86-103) has low GAG affinity. Our laboratory reported dissociation constants (K_D_) of 61 nM [low molecular weight (LMW) heparin], 4.8 nM (HS) and 4.4 nM (DS) for CXCL9(74-103) [22,38]. Interestingly, CXCL9(74-103) showed a 12-fold higher affinity for HS compared with LMW heparin. These data also demonstrate that CXCL9(74-103) binds with a high but different affinity to these GAGs, which confers it a certain degree of binding specificity. In contrast, CXCL9(86-103) demonstrated a reduced affinity for GAGs with a K_D_ of 820 nM for heparin binding. This was also reflected in our results, where CXCL9(86-103) showed no overall effect on angiogenesis, despite seeming to affect FGF-2-induced proliferation. CXCL9(86-103) could not significantly improve diabetes-induced vascular leakage and had also no anti-angiogenic activity in several in vivo models, including tumor-associated angiogenesis and FGF-2-induced angiogenesis in the Matrigel plug assay (data not shown).

Earlier studies that report on targeting HS-mediated interactions most commonly rely on HS mimetics that either mask HS for GF binding or directly interact with the GF, preventing it from binding to HS [39]. For example, LHT7, a chemically modified heparin was shown to inhibit angiogenesis by reducing tyrosine receptor activation by FGF-2 and VEGF165 via direct interaction with the two said growth factors [40]. Pagano et al. reported that the small molecule inhibitor SM27, derived from the FGF-2-binding region of thrombospondin-1 (TSP-1), selectively bound the heparin-binding site of FGF-2 leading to reduced binding to FGFR1 and HS [41]. As such, SM27 inhibited FGF-2-induced angiogenesis by interfering with a ternary complex formation between FGF, FGFR and HS. A computational and chemical strategy was used to investigate derivates of SM27, and several bi-naphtalenic small molecules were identified that bound FGF-2 and inhibited angiogenesis with increased potency over the original molecule [42]. Recently, it was revealed that another TSP with anti-angiogenic activity, TSP-2, was also able to interact with FGF-2 and interfered with HS and FGFR1 binding. These findings indicated that TSP-2 could serve as a lead molecule to further develop FGF-2 inhibitors [43]. In addition, a collagen Vα1-derived fragment with heparin-binding properties was shown to selectively bind to FGF-2, not VEGF-A, and inhibited only the FGF-2-mediated angiogenic processes [44]. Our proposed CXCL9-derived peptide acted both as an HS- and GF-binding molecule. CXCL9(74-103) could potently inhibit VEGF165 binding to HS, which can account for the diminished phosphorylation of ERK by VEGF165 in the presence of CXCL9(74-103). Accordingly, we showed that CXCL9(74-103) interfered with VEGF165-induced endothelial cell proliferation, migration and adhesion. As such, it is plausible that CXCL9(74-103) prevents the formation of a functioning VEGF165:HS:VEGFR2 complex, resulting in inadequate signaling. Spontaneous proliferation was also significantly decreased by the high affinity GAG-binding peptide, which is probably due to inhibition of autocrine growth factors. Moreover, in a mouse model of vascular leakage, in which VEGF165 is thought to be a major contributor, CXCL9(74-103) ameliorated the diabetes-induced vascular permeability. The decrease in blood vessel outgrowth in the cornea after thermal cauterization in CXCL9(74-103)-treated mice, further underlines the effectiveness of the CXCL9-derived peptide to counteract VEGF in vivo.

In addition to binding to HS, CXCL9(74-103) also showed a direct association with FGF-2, with high nanomolar affinity. The obtained K_D_ value lies within the range of the concentrations that were applied in the different in vivo and in vitro assays reported here. FGF-2-mediated proliferation, chemotaxis and sprouting in vitro was diminished by CXCL9(74-103). Also in vivo, CXCL9(74-103) treatment attenuated FGF-2-mediated neovascularization in the Matrigel plug assay. The inhibitory mechanism responsible for countering FGF-2 could be that (1) by direct interaction with FGF-2, CXCL9(74-103) can trap the GF, preventing it from binding to HS and its FGFR or/and that (2) the concomitant binding of CXCL9(74-103) and FGF-2 to HS prevents effective signaling complex formation. Our observation that VEGF165 did not interact with CXCL9(74-103) could be explained by the observation that the structure and topology of the heparin-binding site of VEGF165 differ from that of other known heparin-binding proteins, such as FGF-2 [45]. Furthermore, VEGF165 is also characterized by an 8-fold lower affinity for heparin compared to FGF-2 [46]. 

Interestingly, in addition to functioning as a co-receptor initiating mitogen-activated protein kinase (MAPK) signaling, autonomous cell signaling through syndecans has also been reported [14,47]. Syndecan-4, the most abundant mammalian syndecan, has been described to function as an independent receptor for several heparin-binding growth factors such as FGFs, VEGFs and PDGFs [47]. Upon ligand binding, the HSPGs oligomerize and initiate signaling components such as mammalian target of rapamycin (mTOR), RAC-α serine/threonine-protein kinase (AKT1) and the Rho family of GTPases, which are responsible for cell migration and adhesion [47]. As such, syndecans are, unlike its family member glypicans, characterized by unique structural features. The extracellular domain bears the GAG chains which allows interaction with GAG-binding motifs of growth factors and with the extracellular matrix which enables adhesion. The transmembrane domain assists in efficient oligomerization and the intracellular domain initiates intracellular signaling cascades and recruits intracellular proteins to the cell surface. Therefore, it could also be possible that HS autonomous processes are targeted by CXCL9(74-103), but previous efforts have pinpointed the difficulty to dissect these events from those in cooperation with GFRs. As stated in the introduction, not EGF, but its receptor EGFR relies on interactions with syndecan-4, and integrins α_6_β_4_ and α_3_β_1_ to stimulate migration, survival and invasion [16]. As such, a cooperative interplay between integrins and syndecans facilitates the translation of extracellular signals to the intracellular compartment. Moreover, a binding site in the extracellular domain of syndecan-4 spanning amino acids 87-131 captures the EGFR and α_3_β_1_ integrin and establishes a direct interaction between EGFR1 and syndecan-4. The cytoplasmic COOH-terminus of syndecan-4 engages with the COOH-terminal part of α_6_β_4_ integrin which in turn causes the cytoplasmic domain of α_6_β_4_ integrin to become phosphorylated and initiate downstream signaling. This led to the development of a peptide based on the EGFR interaction motifs, called synstatin (SSTN_EGFR_) that displaces the RTK and α_3_β_1_ integrin from the syndecan and prevents cell migration [48]. We showed that EGF-mediated endothelial cell proliferation, migration and sprouting were inhibited by CXCL9(74-103), including phosphorylation of ERK. Therefore, it is plausible that CXCL9(74-103) inhibits EGF-mediated processes by binding to the HS chains of endothelial syndecans, thereby interrupting syndecan:integrin:EGFR complex formation. This idea is strengthened by the observation that inhibition of EGF-induced endothelial sprouting by CXCL9(74-103) is abolished when the expression of cell surface HS is reduced. Also in vivo, CXCL9(74-103) attenuated angiogenesis in a breast tumor model wherein EGFR is upregulated. Similarly, in triple-negative breast cancer patients, an association between syndecan-1 and EGFR overexpression has been found [49]. Upon syndecan-1 depletion, EGFR expression, as well as EGF-induced activation of Akt was reduced. In addition to EGF, a crosstalk between syndecans, integrin α_v_β_3_ and VEGF in neovascularization has also been proposed. The used substratum gelatin in the adhesion assay also contains the RGD cell adhesive motif as a main receptor recognition pattern, which binds several integrins, but primarily α_5_β_1_ and α_v_β_3_ integrins [50]. Moreover, α_5_β_1_ and α_v_β_3_ are two of the three integrins that mainly synergize with syndecans in the regulation of angiogenesis [14]. Therefore, the disruption of an integrin:syndecan:VEGFR2 complex could also explain the CXCL9(74-103)-induced inhibition of endothelial cell adhesion in response to VEGF165. 

Finally, we conclude that CXCL9(74-103) shows clear anti-angiogenic activity in vitro and in vivo by interfering with HSPG-mediated growth factor signaling. CXCL9(74-103) was able to counter various growth factor (EGF, VEGF165, FGF-2)-induced effects via different mechanisms. Considering the widespread involvement of HSPGs in different processes either autonomous or in conjunction with other players, difficulties arise to pinpoint the exact mechanism of action of CXCL9(74-103). However, more importantly, current anti-angiogenic cancer therapies targeting VEGF-A are confronted with resistance due to upregulation of other growth factors, such as FGF-2, EGF and PDGF by tumor cells. Therefore, strategies that target multiple growth factors can possibly subvert such compensatory mechanisms. In conclusion, CXCL9(74-103) forms an interesting lead molecule to further develop anti-angiogenics. 

## Data Availability

All data are available on request from the authors.

## References

[1] Rajabi M., Mousa S.A. (2017). The Role of Angiogenesis in Cancer Treatment. Biomedicines.

[2] Ellis L.M. (2004). Epidermal growth factor receptor in tumor angiogenesis. Hematol. Clin. N. Am..

[3] Babina I.S., Turner N.C. (2017). Advances and challenges in targeting FGFR signalling in cancer. Nat. Rev. Cancer.

[4] Claesson-Welsh L., Welsh M. (2013). VEGFA and tumour angiogenesis. J. Intern. Med..

[5] Elfenbein A., Simons M. (2010). Auxiliary and Autonomous Proteoglycan Signaling Networks. Methods Enzymol..

[6] Iozzo R.V., Schaefer L. (2015). Proteoglycan form and function: A comprehensive nomenclature of proteoglycans. Matrix Biol. J. Int. Soc. Matrix Biol..

[7] Afratis N., Gialeli C., Nikitovic D., Tsegenidis T., Karousou E., Theocharis A.D., Pavao M.S.G., Tzanakakis G., Karamanos N.K. (2012). Glycosaminoglycans: Key players in cancer cell biology and treatment. FEBS J..

[8] Schaefer L., Schaefer R.M. (2010). Proteoglycans: From structural compounds to signaling molecules. Cell Tissue Res..

[9] Nagarajan A., Malvi P., Wajapeyee N. (2018). Heparan Sulfate and Heparan Sulfate Proteoglycans in Cancer Initiation and Progression. Front. Endocrinol..

[10] van Wijk X.M., van Kuppevelt T.H. (2014). Heparan sulfate in angiogenesis: A target for therapy. Angiogenesis.

[11] Schlessinger J., Plotnikov A.N., Ibrahimi O.A., Eliseenkova A.V., Yeh B.K., Yayon A., Linhardt R.J., Mohammadi M. (2000). Crystal Structure of a Ternary FGF-FGFR-Heparin Complex Reveals a Dual Role for Heparin in FGFR Binding and Dimerization. Mol. Cell.

[12] Tkachenko E., Rhodes J.M., Simons M. (2005). Syndecans: New kids on the signaling block. Circ. Res..

[13] Cecchi F., Pajalunga D., Fowler C.A., Üren A., Rabe D.C., Peruzzi B., MacDonald N.J., Blackman D.K., Stahl S.J., Byrd R.A. (2012). Targeted Disruption of Heparan Sulfate Interaction with Hepatocyte and Vascular Endothelial Growth Factors Blocks Normal and Oncogenic Signaling. Cancer Cell.

[14] Afratis N., Nikitovic D., Multhaupt H.A.B., Theocharis A.D., Couchman J.R., Karamanos N.K. (2017). Syndecans—Key regulators of cell signaling and biological functions. FEBS J..

[15] Morgan M., Humphries M.J., Bass M.D. (2007). Synergistic control of cell adhesion by integrins and syndecans. Nat. Rev. Mol. Cell Biol..

[16] Wang H., Jin H., Beauvais D.M., Rapraeger A.C. (2014). Cytoplasmic Domain Interactions of Syndecan-1 and Syndecan-4 with α6β4 Integrin Mediate Human Epidermal Growth Factor Receptor (HER1 and HER2)-dependent Motility and Survival. J. Biol. Chem..

[17] Hassan N., Greve B., Espinoza-Sánchez N.A., Götte M. (2021). Cell-surface heparan sulfate proteoglycans as multifunctional integrators of signaling in cancer. Cell. Signal..

[18] Fuster M.M., Wang L., Castagnola J., Sikora L., Reddi K., Lee P.H., Radek K.A., Schuksz M., Bishop J.R., Gallo R.L. (2007). Genetic alteration of endothelial heparan sulfate selectively inhibits tumor angiogenesis. J. Cell Biol..

[19] Russo M., Giavazzi R. (2018). Anti-angiogenesis for cancer: Current status and prospects. Thromb. Res..

[20] Ronca R., Benkheil M., Mitola S., Struyf S., Liekens S. (2017). Tumor angiogenesis revisited: Regulators and clinical implications. Med. Res. Rev..

[21] Jayson G.C., Hicklin D.J., Ellis L.M. (2012). Antiangiogenic therapy—evolving view based on clinical trial results. Nat. Rev. Clin. Oncol..

[22] Vanheule V., Janssens R., Boff D., Kitic N., Berghmans N., Ronsse I., Kungl A.J., Amaral F.A., Teixeira M.M., Van Damme J. (2015). The Positively Charged COOH-terminal Glycosaminoglycan-binding CXCL9(74–103) Peptide Inhibits CXCL8-induced Neutrophil Extravasation and Monosodium Urate Crystal-induced Gout in Mice. J. Biol. Chem..

[23] Vanheule V., Boff D., Mortier A., Janssens R., Petri B., Kolaczkowska E., Kubes P., Berghmans N., Struyf S., Kungl A.J. (2017). CXCL9-Derived Peptides Differentially Inhibit Neutrophil Migration In Vivo through Interference with Glycosaminoglycan Interactions. Front. Immunol..

[24] Boff D., Crijns H., Janssens R., Vanheule V., Menezes G.B., Macari S., Silva T.A., Amaral F.A., Proost P. (2018). The chemokine fragment CXCL9(74-103) diminishes neutrophil recruitment and joint inflammation in antigen-induced arthritis. J. Leukoc. Biol..

[25] Vanheule V., Crijns H., Poosti F., Ruytinx P., Berghmans N., Gerlza T., Ronsse I., Pörtner N., Matthys P., Kungl A.J. (2018). Anti-inflammatory effects of the GAG-binding CXCL9(74-103) peptide in dinitrofluorobenzene-induced contact hypersensitivity in mice. Clin. Exp. Allergy.

[26] Van Raemdonck K., Berghmans N., Vanheule V., Bugatti A., Proost P., Opdenakker G., Presta M., Van Damme J., Struyf S. (2014). Angiostatic, tumor inflammatory and anti-tumor effects of CXCL4(47-70) and CXCL4L1(47–70) in an EGF-dependent breast cancer model. Oncotarget.

[27] Abu El-Asrar A.M., Ahmad A., Siddiquei M.M., De Zutter A., Allegaert E., Gikandi P.W., De Hertogh G., Van Damme J., Opdenakker G., Struyf S. (2019). The Proinflammatory and Proangiogenic Macrophage Migration Inhibitory Factor Is a Potential Regulator in Proliferative Diabetic Retinopathy. Front. Immunol..

[28] García-Caballero M., Paupert J., Blacher S., Van de Velde M., Quesada A.R., Medina M.A., Noël A. (2017). Targeting VEGFR-3/-2 signaling pathways with AD0157: A potential strategy against tumor-associated lymphangiogenesis and lymphatic metastases. J. Hematol. Oncol..

[29] Hamidi H., Lilja J., Ivaska J. (2017). Using xCELLigence RTCA Instrument to Measure Cell Adhesion. Bio-Protocol.

[30] Zahra F.T., Choleva E., Sajib S., Papadimitriou E., Mikelis C.Μ. (2019). In Vitro Spheroid Sprouting Assay of Angiogenesis. Breast Cancer.

[31] Hafezi-Moghadam A., Tombran-Tink J., Barnstable C.J., Gardner T.W. (2012). Mechanisms of blood–retinal barrier breakdown in diabetic retinopathy. Visual Dysfunction in Diabetes: The Science of Patient Impairment and Health Care.

[32] Klaassen I., Van Noorden C.J., Schlingemann R.O. (2013). Molecular basis of the inner blood-retinal barrier and its breakdown in diabetic macular edema and other pathological conditions. Prog. Retin. Eye Res..

[33] Philipp W., Speicher L., Humpel C. (2000). Expression of vascular endothelial growth factor and its receptors in inflamed and vascularized human corneas. Investig. Ophthalmol. Vis. Sci..

[34] Saika S. (2004). TGF-beta signal transduction in corneal wound healing as a therapeutic target. Cornea.

[35] Yoshida S., Yoshida A., Matsui H., Takada Y.I., Ishibashi T. (2003). Involvement of macrophage chemotactic protein-1 and interleukin-1beta during inflammatory but not basic fibroblast growth factor-dependent neovascularization in the mouse cornea. Lab. Investig..

[36] Hossein-Nejad-Ariani H., AlThagafi E., Kaur K. (2019). Small Peptide Ligands for Targeting EGFR in Triple Negative Breast Cancer Cells. Sci. Rep..

[37] Johns S.C., Yin X., Jeltsch M., Bishop J.R., Schuksz M., El Ghazal R., Wilcox-Adelman S.A., Alitalo K., Fuster M.M. (2016). Functional Importance of a Proteoglycan Coreceptor in Pathologic Lymphangiogenesis. Circ. Res..

[38] Vanheule V., Vervaeke P., Mortier A., Noppen S., Gouwy M., Snoeck R., Andrei G., Van Damme J., Liekens S., Proost P. (2016). Basic chemokine-derived glycosaminoglycan binding peptides exert antiviral properties against dengue virus serotype 2, herpes simplex virus-1 and respiratory syncytial virus. Biochem. Pharmacol..

[39] Chiodelli P., Bugatti A., Urbinati C.E., Rusnati M. (2015). Heparin/Heparan Sulfate Proteoglycans Glycomic Interactome in Angiogenesis: Biological Implications and Therapeutical Use. Molecules.

[40] Chung S.W., Bae S.M., Lee M., Al-Hilal T.A., Lee C.K., Kim J.K., Kim I.-S., Kim S.Y., Byun Y. (2015). LHT7, a chemically modified heparin, inhibits multiple stages of angiogenesis by blocking VEGF, FGF2 and PDGF-B signaling pathways. Biomaterials.

[41] Pagano K., Torella R., Foglieni C., Bugatti A., Tomaselli S., Zetta L., Presta M., Rusnati M., Taraboletti G., Colombo G. (2012). Direct and Allosteric Inhibition of the FGF2/HSPGs/FGFR1 Ternary Complex Formation by an Antiangiogenic, Thrombospondin-1-Mimic Small Molecule. PLoS ONE.

[42] Foglieni C., Pagano K., Lessi M., Bugatti A., Moroni E., Pinessi D., Resovi A., Ribatti D., Bertini S., Ragona L. (2016). Integrating computational and chemical biology tools in the discovery of antiangiogenic small molecule ligands of FGF2 derived from endogenous inhibitors. Sci. Rep..

[43] Rusnati M., Borsotti P., Moroni E., Foglieni C., Chiodelli P., Carminati L., Pinessi D., Annis D.S., Paiardi G., Bugatti A. (2018). The calcium-binding type III repeats domain of thrombospondin-2 binds to fibroblast growth factor 2 (FGF2). Angiogenesis.

[44] Jia T., Vaganay E., Carpentier G., Coudert P., Guzman-Gonzales V., Manuel R., Eymin B., Coll J.-L., Ruggiero F. (2020). A collagen Vα1-derived fragment inhibits FGF-2 induced-angiogenesis by modulating endothelial cells plasticity through its heparin-binding site. Matrix Biol..

[45] Fairbrother W.J., Champe M.A., Christinger H.W., Keyt B.A., Starovasnik M.A. (1998). Solution structure of the heparin-binding domain of vascular endothelial growth factor. Structure.

[46] Cochran S., Li C.P., Ferro V. (2008). A surface plasmon resonance-based solution affinity assay for heparan sulfate-binding proteins. Glycoconj. J..

[47] Elfenbein A., Simons M. (2013). Syndecan-4 signaling at a glance. J. Cell Sci..

[48] Wang H., Jin H., Rapraeger A.C. (2015). Syndecan-1 and syndecan-4 capture epidermal growth factor receptor family members and the α3β1 integrin via binding sites in their ectodomains: Novel synstatins prevent kinase capture and inhibit α6β4-integrin-dependent epithelial cell motility. J. Biol. Chem..

[49] Ibrahim S.A., Gadalla R., El-Ghonaimy E.A., Samir O., Mohamed H.T., Hassan H., Greve B., El-Shinawi M., Mohamed M.M., Götte M. (2017). Syndecan-1 is a novel molecular marker for triple negative inflammatory breast cancer and modulates the cancer stem cell phenotype via the IL-6/STAT3, Notch and EGFR signaling pathways. Mol. Cancer.

[50] Davidenko N., Schuster C.F., Bax D.V., Farndale R.W., Hamaia S., Best S.M., Cameron R.E. (2016). Evaluation of cell binding to collagen and gelatin: A study of the effect of 2D and 3D architecture and surface chemistry. J. Mater. Sci. Mater. Med..

